# Supplementary material for: A non-methanogenic archaeon within the order Methanocellales
Source: Nat Commun. 2024 Jun 13;15:4858. doi: 10.1038/s41467-024-48185-5 (PMC11176372; doi:10.1038/s41467-024-48185-5)
Supplement: Supplementary file 1 — Supplementary information [file 41467_2024_48185_MOESM1_ESM.pdf]

SUPPORTING INFORMATION

Supplementary Note 1. Genomic completeness of the MAG Met12

The genome size of the circularized MAG Met12 was 1,067,436 bp, which was the smallest within both orders of Methanocellales and Methanosarcinales (Fig. 2a). The circularized MAG Met12 was confirmed to have all tRNA for the usage of twenty amino acids and one set of rRNA gene island including 5S rRNA, 16S rRNA, and 23S rRNA. The genome completeness was 92.81% and the contamination was 0%, both of which were assessed by CheckM v1.1.3[1]. Marker lineage of p_Euryarchaeota (UID49) on the CheckM algorithm was used for the assessment of the genome completeness, and twelve Pfam/TIGRfam (PF00521, PF00986, PF03989, PF01269, PF01798, PF03684, PF04242, PF04312, PF04414, PF06550, PF09202, TIGR00050) out of 228 marker proteins were absent in the MAG Met12. Within these proteins, GyrABC (PF00521, PF00986, and PF03989), which encodes a type II topoisomerase (DNA gyrase), was also identified as absent from the genome of the closely related *Ca.* Methanoflorens stordalmirensis. In addition, three proteins of PF04312 (DUF460), PF09202 (Rio2, N-terminal), and PF01798 (snoRNA binding domain, fibrillarin) were absent in the one or two close relative genomes of Met12, suggesting that the absence of those marker proteins was potentially the original feature of this lineage. The absence of the remaining six marker proteins (PF01269, PF03684, PF04242, PF04414, PF06550, TIGR00050) was unique to the MAG of Met12. Within those six marker proteins, the PF03684 and PF04242 are unknown function proteins. The PF01269 and TIGR00050 are involved in RNA methylation, the PF04414 encodes D-aminoacyl-tRNA deacylases which keeps chiral errors away from the translational machinery, and the PF06550 encodes signal-peptide peptidase, presenilin aspartyl protease. It is presumed that these are not essential proteins, thus may have been lost the genes from the Met12 genome during via genome streamlining.

To further validate the completeness of MAG Met12, genes associated with the primary biosynthesis pathways were analyzed. The Met12 genome encoded the entire DNA biosynthesis pathway (pyrimidine metabolism, ko00240 and purine metabolism, ko00230) and encoded all amino acid biosynthesis pathways (ko01230) except for cysteine biosynthesis. The absence of cysteine biogenesis was also a common feature within the group of close relatives such as *Ca.* Methanoperedens, *Ca.* Syntrophoarchaeum, and Methanocella; thus, cysteine must be provided from the others for this clade of Archaea. Regarding the biosynthesis of co-factors, no genes for biosynthesis of CoM, CoB, and F_{430} were present in the MAG Met12 (Fig.2a), but genes for the biosynthesis of F_{420} (cofCDEGH), methanofuran (mfnABDEF), molybdopterin (moaACDE), heme (M00121), thiamine (M00127), NAD (M00115), cobalamin (M00122), and riboflavin (M00125) were present in the MAG Met12 (Supplementary Data 1). Based on these features of the genome constitution, the circularized MAG of Met12 has no significant defects in the biogenesis pathways except for co-factors associated with methanogenesis.

Supplementary Note 2. Taxonomic analysis of the MAG Met12

The 16S rRNA sequence of the MAG Met12 was confirmed to be identical to the Ced_A1 (KC574884) which was obtained from the 16S rRNA (full-length) based taxonomic

analysis of The Cedars springs[2]. The 16S rRNA surveys of the various serpentinized ecosystems reported that the Ced_A1 related organisms (corresponding to Met12) were distributed in terrestrial and oceanic serpentinization sites and the taxon containing these organisms was then named The Cedars Methanosarcinales (TCMS)[3]. The SILVA database[4] that is based on the 16S rRNA gene placed Met12 in the phylum Halobacteriota, class Methanosarcinia, order Methanosarcinales, family Syntrophoarchaeaceae, while the Genome Taxonomy Database (GTDB)[5] classification placed it in the order Methanocellales. In this study, we adopted the result of GTDB as the taxonomic classification of Met12, but further consideration may be required as more Met12-related genomic information becomes available.

Supplementary Note 3. Validation of lack of Mcr, Mtr, and Hdr in the MAG Met12

Given that Mcr and Mtr, two essential protein complexes for the final step of methanogenesis, and Hdr, an essential protein complex for the electron bifurcation for reducing ferredoxin, are highly conserved in the orders Methanocellales and Methanosarcinales the lack of these key genes was further evaluated. Since MAG Met12 was extracted from the metagenome, we evaluated the confidence in the absence of these methanogenic genes in MAG Met12 based on the presence (absence) of major methanogenic genes in the metagenomes and their read coverages. BS5 pool and BS5sc metagenomic data used to recover MAG Met12 contained two archaea, Methanocellales archaeon Met12 (coverage of 280) and Methanobacterium archaeon Met13 (coverage of 11) (Supplementary Fig. 1b). However, whole metagenome analysis of BS5sc revealed that 1) only one gene island encoding *mtrHGFABCDE-mcrAGCDB* was present in the data set, 2) the coverage of that island was 14, and 3) no other *mcr* or *mtr* genes were detected in the other BS5sc contigs. Similar results were observed in the metagenomes of the BS5 pool for both 2011 and 2012, indicating that the MAG Met12 did not encode Mcr and Mtr.

As for the Hdr, no *hdrDE* genes were detected in the BS5sc and BS5 pool metagenomes, indicating that Met12 does not encode HdrDE. As for *hdrABC*, three contigs in the BS5sc metagenomic dataset encoded the *hdrABC* genes. The three contigs were detected at about 1000, 60, and 11 read coverage, respectively, which corresponds to the read coverage of MAG Chloroflexi bacterium Chl2, MAG *Ca.* Lithacetigenota Unc11, and MAG Met13[6,7] in the BS5sc metagenome. No *hdrABC* gene island was detected at about 280 read coverage, which corresponds to the MAG Met12. Similar trends were revealed for the Hdr in the BS5 pool metagenomes. Based on these metagenomic analyses, we concluded that the Met12 has a unique genomic constitution lacking *mcr*, *mtr*, and *hdr* as well as the associated co-factors biosynthetic pathways.

Supplementary Note 4. Further discussions of *in situ* gene expression analysis:

1) Multi-heme c-type cytochrome, MmcX

The MmcX gene coding 4-heme *c*-type cytochrome showed the highest expression in The Cedars springs (Fig. 3). Multi-heme *c*-type cytochromes (MHcytCs) have been widely recognized for their importance in both archaeal methanogenesis and anaerobic methane oxidation. A membrane-bound 6-heme *c*-type cytochrome MmcA in the *Methanosarcina acetivorans* C2A functions in the electron-donating reaction to soluble electron shuttle, anthraquinone-2,6-disulfonate (AQDS)[8], and in the electron-accepting reaction in the direct

interspecies electron transfer (DIET) between the electron-releasing *Geobacter metallireducens* and the *M. acetivorans* C2A[9]. These studies indicated that the MmcA has a capacity for both donating and accepting electrons. Meanwhile, anaerobic methane-oxidizing archaea in the order *Methanosarcinales* are also known to use MHcytC. In the ANME reaction, MHcytCs containing tens of hemes (MHCs) is responsible for the DIET reaction to export electrons to the EET-capable and respirable bacterial partners, like sulfate reducers[10,11]. While five different MHcytCs were identified in the MAG Met12, none of them showed high similarity to either the MmcA or the MHCs based on the orthologous analysis (Fig. 2a, Supplementary Data 1).

The orthologous analysis of MmcX showed that the orthologues of the MmcX are mainly encoded by the members in the order Methanosarcinales (Fig. 3a, Supplementary Fig. 3, Supplementary Table 4). The MmcX encoded by hyperthermophilic *Ferroglobus placidus* (Ferp_0668) was highly expressed during the respiration coupled with solid-state iron reduction[12], and the MmcX in an anaerobic alkane-degrading archaeon, *Ca.* Syntrophoarchaeum butanivorans (SBU_000777), was highly expressed during syntrophic butane degradation via DIET between the archaeon and the sulfate-reducing bacterium[13]. While the heterologous expression of MmcX in Met12 suggested that it has an electron-accepting capacity, the Ferp_0668 in *F. placidus* and SBU_000777 in *Ca.* S. butanivorans presume to donate an electron to the oxidized compounds. Namely, the direction of the extracellular electron transfer reaction of the MmcX orthologues in the *F. placidus* and *Ca.* S. butanivorans is the opposite direction than that of the MmcX in the Met12. These studies on the orthologous proteins of MmcX imply that MHcytCs have the ability to adapt to the various reactive redox potentials, and in the ultra-reducing setting where electrons donors are readily available but electron acceptors are not, MmcX could be tuned to import electrons at the lower redox range.

Recently, the crystal structures of two archaeal extracellular cytochrome nanowires (ECNs) from hyperthermophilic archaea, *Pyrobaculum calidifontis* and *Archaeoglobus veneficus*, were determined using cryo-EM[14]. The ECN from *Archaeoglobus veneficus* (AvECN) was identified as the orthologue of MmcX in the Met12. To assess whether MmcX is a potential nanowire, we compared the structure of the signal peptide region of AvECN and MmcX. Notably, the structures of the N-terminal transmembrane region and the cleavage site were identical, indicating a similar localization for both proteins (Supplementary Fig. 5). This strongly suggests that MmcX is also likely a nanowire protein.

2) Archaeal pilin

The second highly expressed gene in the MAG Met12 (ATZ60936.1) encodes a predicted archaea-type pili (arCOG06141) (Fig. 3). General function of pili is involved in attachment to a solid surface, biofilm formation, pathogenicity, or microbial conjugation[15]. Recent studies have reported that solid iron-reducing microbes form conductive pili (e-Pili) as an important element for extracellular electron transfer to the solid surfaces[16]. The conductivity of the potential e-pili could be estimated from % of the aromatic amino acid (FWHY) contents per pilin[17]. Overall densities of the aromatic amino acids in PilA of cable bacteria, electrically conductive multicellular filamentous bacteria, are 7 to 10%, and those of other e-pili in electroactive Geobacteraceae bacteria are about 9 to 15%[18]. The density of aromatic amino acids of the pilin of Met12 is 7.93%, indicating that the pilin of Met12 is potentially an e-pili but may be more critical in the function of attachment.

3) Other highly expressed genes

In addition to the these two highly expressed genes (MmcX and Pilin), a group of genes encoding CRISPR and minimal nucleotidyltransferase (K07076 and K07075, or arCOG01195) were highly expressed in the environment (Supplementary Data 2). CRISPR contains tens of base pairs of short repeats and acts as an immune system in prokaryotes, which contributes to resistance to foreign nucleotide invasions such as plasmids, phages, and viruses. Fifteen copies of the short repeat sequence (ATTTCAATCCCACTATGGTCTGATTTTAAT) were detected on the MAG Met12, and the sequences were found to be widely distributed in the CRISPR region of archaeal genomes. Since the study of *Methanosarcina mazei* reported that high expression of CRISPR was also induced by the high salinity stress of NaCl[19], the repeat sequences in the MAG Met12 could be important for the immunity and/or the environmental stresses.

Minimal nucleotidyltransferases were also conserved enzymes that are present in the genomes of archaea[20]. The high expression of these genes suggests that the defense or repair of DNA damage is necessary to live in the high pH environment of The Cedars.

In addition, ribosomal proteins and V-type ATP synthase (*ntpA-H*) were highly expressed, suggesting that Met12 is actively engaged in protein synthesis and ATP production. Given that Met12 does not encode the Mcr and Mtr complexes, it cannot generate a Na^+ gradient in the usual way, and generation of membrane potential for ATP synthesis may involve membrane-bound Rnf complexes and Mrp transporters (Fig. 2b, Fig. 5c).

Within the acetyl-CoA pathway, the genes for CODH complex (*cdhA-E*) showed relatively high expression. CODH is the enzyme that produces acetyl-CoA, which is an essential metabolite for biosynthesis and substrate-level ATP synthesis associated with acetogenesis (Fig. 2b). High expression of CODH indicated that acetogenesis was occurring in the Met12 at the serpentinized setting.

Supplementary Note 5. Evolutionary perspective of methanogenic pathway

Since a methanogenic pathway is highly conserved within the traditional seven orders of the super phylum Euryarchaeota (Methanobacteriales, Methanococcales, Methanomicrobiales, Methanosarcinales, Methanocellales, Methanopyrales and Methanomassiliicoccales), it is not clear why Met12 (in the order Methanocellales) lacks methyl-coenzyme M reductase (MCR), the essential gene required for methanogenesis.

Several *Archaeoglobus* species in the superphylum Euryarchaeota and MAGs in the phylum Bathyarchaeota harbor archaeal acetyl-CoA pathway (Fwd/Fmd, Ftr, Mch, Mtd, Mer) but lack the MCR complex[21,22]. Meanwhile, the phylum Verstraetearchaeota lacks the archaeal acetyl-CoA pathway but harbors the MCR complex and is presumed to produce methane from methoxy compounds[23]. Considering such a versatile pathway, the acetyl-CoA pathway and the MCR complex should evolve distinctly, and the genomic constitutions of Met12-like MAGs may reflect the trace of the evolutionary process of the methanogenic pathway in the traditional seven orders of methanogenic Euryarchaeota. Other than that, the lack of methanogenic capabilities in Met12 may result from genome streamlining under extreme conditions with a low concentration of bioavailable inorganic carbon and phosphate at The Cedars setting[2].

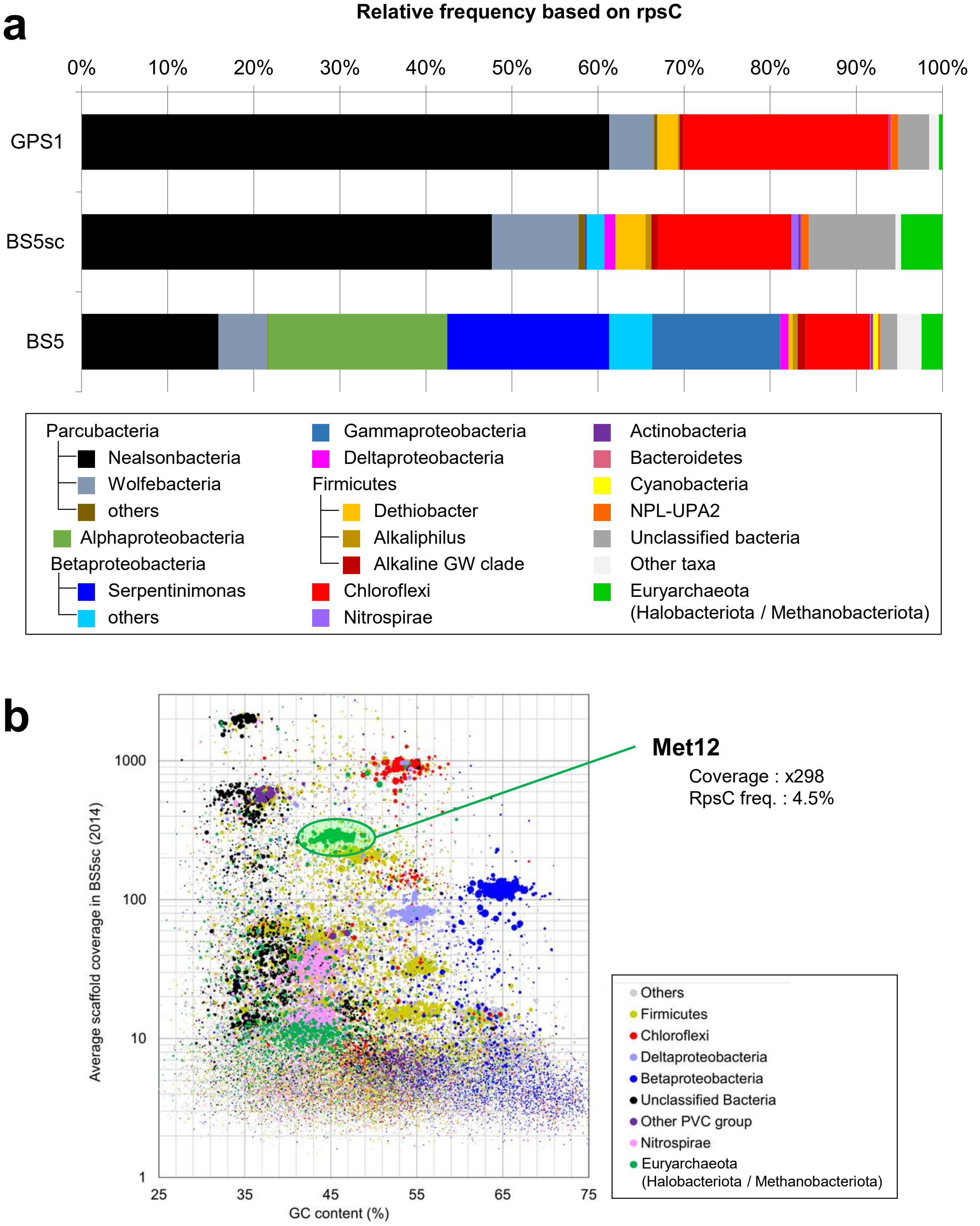

Supplementary Fig. 1. Microbial community composition within The Cedars serpentinization groundwater and MAG of Met12 in the BS5sc metagenome.

a, Comparison of microbial community compositions based on Phylum or Class taxa of RpsC gene from the metagenomic analysis. As for Parcubacteria, Betaproteobacteria and Firmicutes, relative frequencies of the lower level of the taxa are shown. Newly named phyla Halobacteriota and Methanobacteriota are shown as phylum Euryarchaeota since the scaffold taxonomy was assigned by GhostKOALA based on NCBI taxonomy. **b,** An average coverage versus G+C content plot for clustering metagenome-assembled genomes (MAGs) from The Cedars spring BS5sc. MAGs were identified using the estimated taxonomic classification (color of dots), length (size of dots), G+C content and mean coverage of scaffolds. MAG Met12 was marked as green circles. The similar plots for The Cedars springs GPS1 and BS were shown in the previous study (Suzuki *et al.* 2017. ISMEJ 11: 2584-2598).

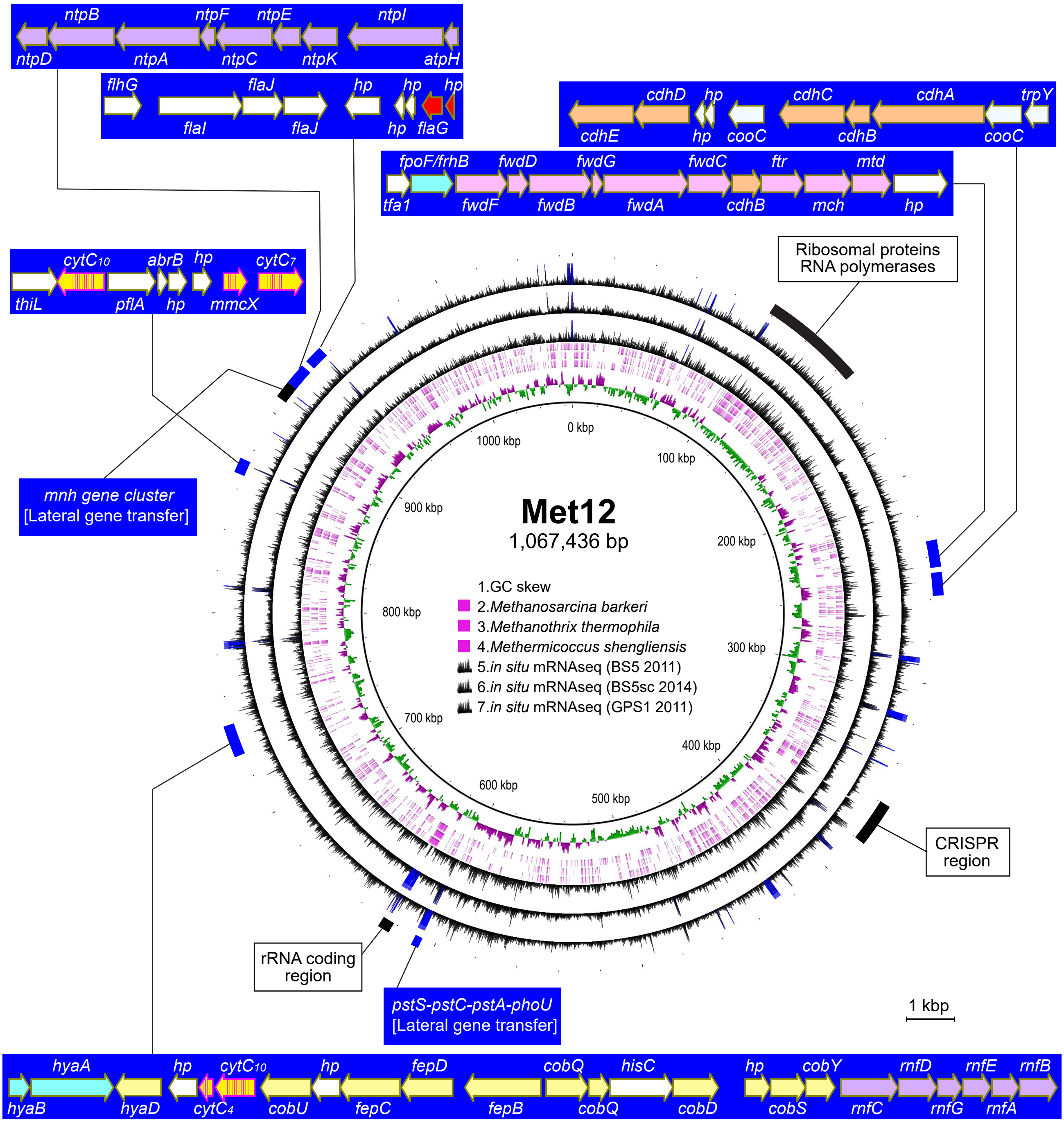

Supplementary Fig. 2. Circulated MAG of Met12 and gene expression profiles.

The inner scales designate the coordinates (in kbp). The first (inner-most) circle shows the GC-skew. The second, third and fourth circles show the blastn analysis against the genomic sequences of Methanosarcina barkeri, Methanothrix thermophila, and Methermicoccus shengliensis, respectively. The fifth, sixth, and seventh circles show the gene expression level in BS5pool (2011), BS5sc (2014), and GPS1 (2011), respectively. The locations of some genes of interest are indicated, which encodes multi-heme c-type cytochromes (MHcytC), central pathway of methanogenesis, ATP synthase, flagellum, etc.

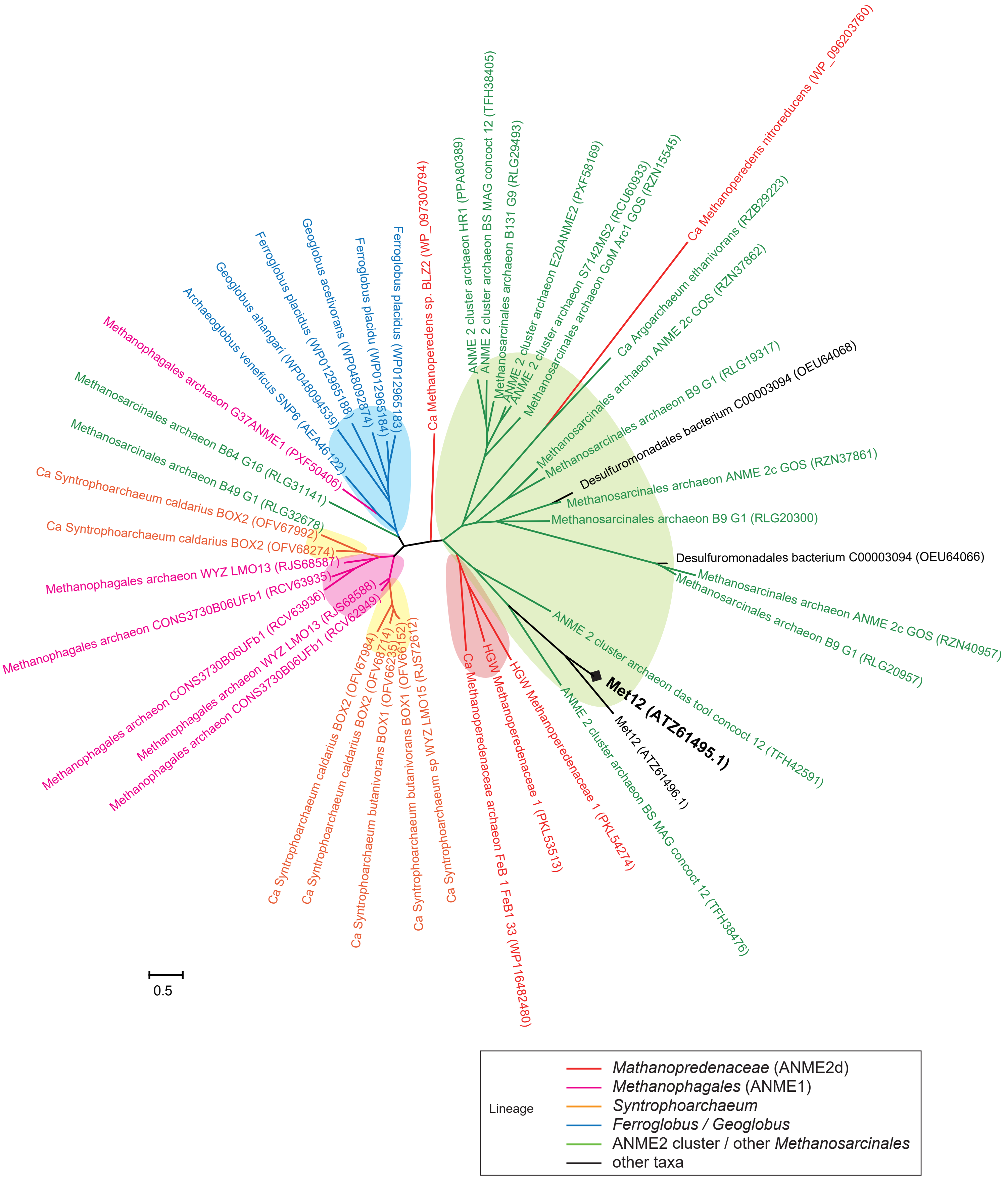

Supplementary Fig. 3. Phylogenetic tree of 4-heme *c*-type cytochrome (MmcX) proteins.

Phylogenetic tree of 4-heme *c*-type cytochrome, MmcX (Fig. 4a) with accession number and strain name of the protein sequences.

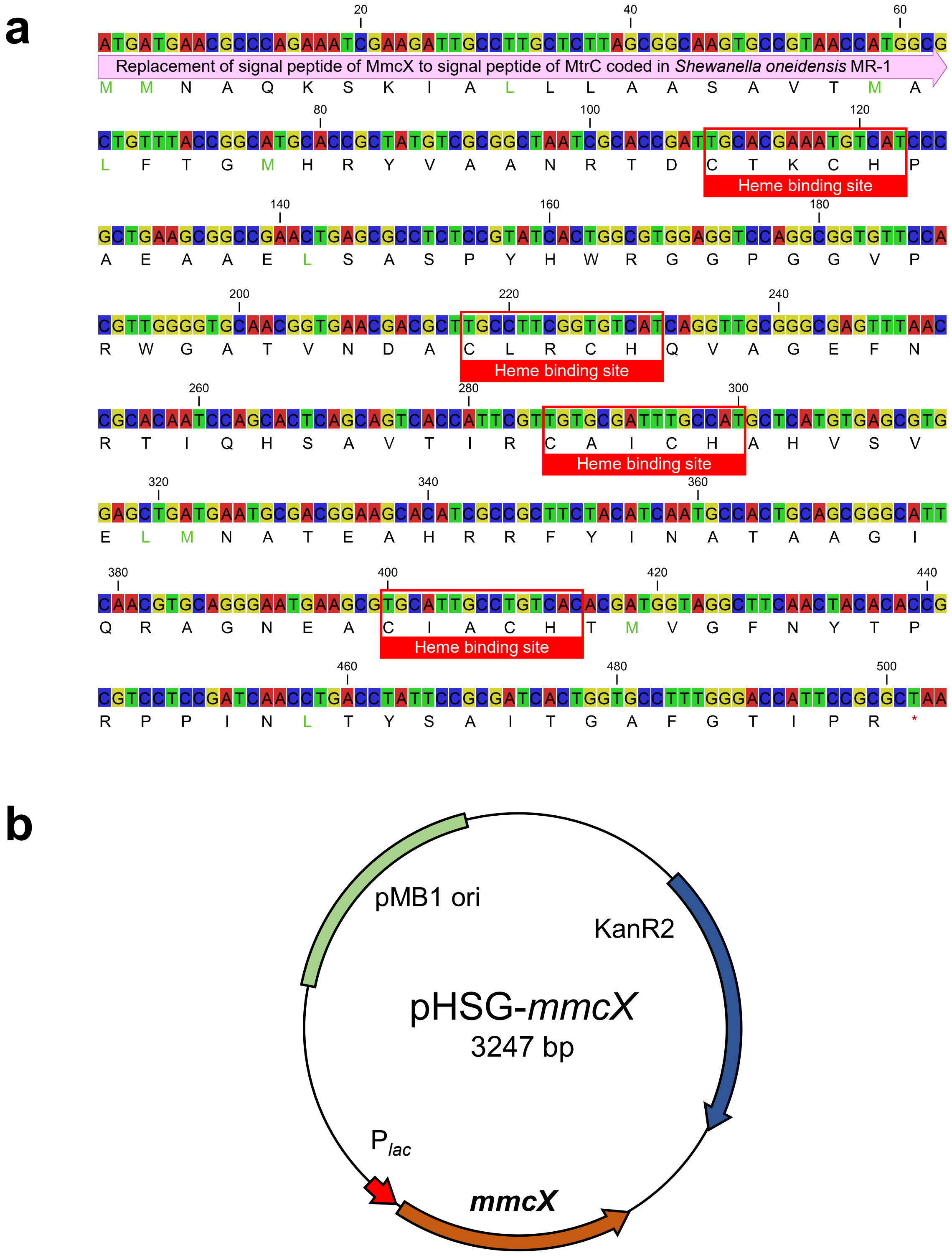

Supplementary Fig. 4. Modified sequence of *mmcX* for the expression analysis in *Shewanella oneidensis* MR-1 and its expression vector.

a, A modified nucleotide sequence of *mmcX* gene for matching to the codon usage for Gammaproteobacteria (*Escherichia coli*). Before modification, signal peptide of MmcX in MAG Met12 (5’-MKNKTLVLMVAVVCIGLFVLPEALA) was replaced to the signal peptide of MtrC coded in *Shewanella oneidensis* MR-1. Four heme binding motifs (CXXCH) are shown in red boxes. **b,** The construction of the expression vector (pHSG-*mmcX*) used in this study.

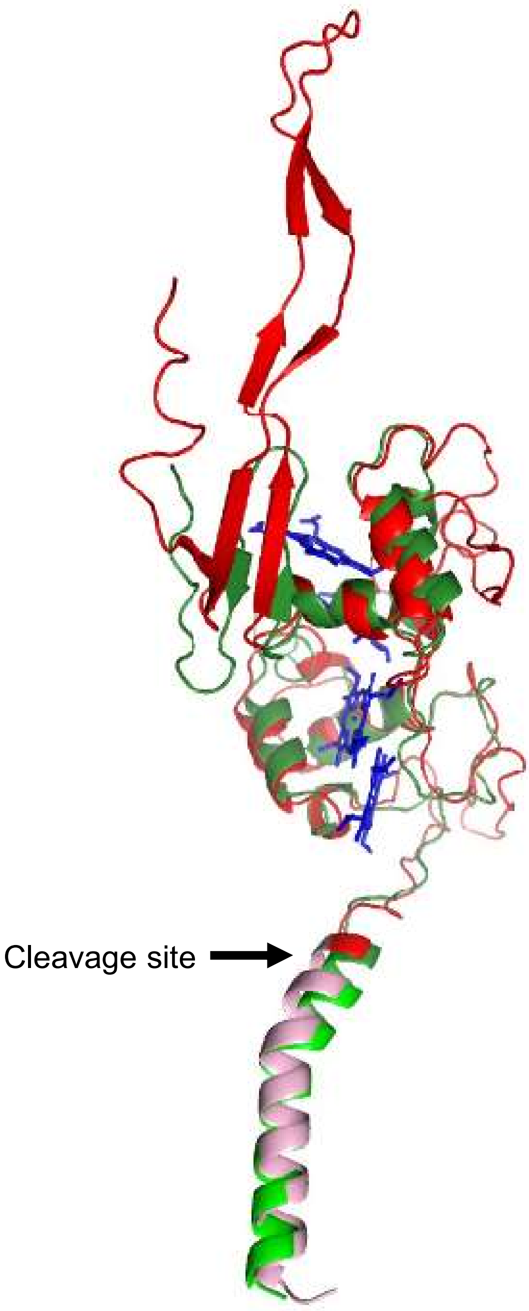

Supplementary Fig. 5. Comparison of predicted crystal structures between MmcX of Met12 and avECN of *Archaeoglobus veneficus*.

The monomeric structures of an archaeal nanowire avECN (in red) and MmcX (in green) were shown as ribbon diagrams. The lightly shaded regions indicate the signal peptide areas, while the cleavage site was indicated by an arrow.

SUPPLEMENTARY REFERENCES

1 Parks, D. H., Imelfort, M., Skennerton, C. T., Hugenholtz, P. & Tyson, G. W. CheckM: assessing the quality of microbial genomes recovered from isolates, single cells, and metagenomes. *Genome Res* **25**, 1043-1055 (2015).

2 Suzuki, S. *et al.* Microbial diversity in The Cedars, an ultrabasic, ultrareducing, and low salinity serpentinizing ecosystem. *Proc Natl Acad Sci USA* **110**, 15336-15341 (2013).

3 Frouin, E. *et al.* Diversity of rare and abundant prokaryotic phylotypes in the Prony hydrothermal field and comparison with other serpentinite-hosted ecosystems. *Front Microbiol* **9**, 102 (2018).

4 Quast, C. *et al.* The SILVA ribosomal RNA gene database project: improved data processing and web-based tools. *Nuc Acid Res* **41**, D590-596 (2013).

5 Chaumeil, P. A., Mussig, A. J., Hugenholtz, P. & Parks, D. H. GTDB-Tk: a toolkit to classify genomes with the Genome Taxonomy Database. *Bioinformatics* **36,** 1925–1927 (2019).

6 Suzuki, S. *et al.* Unusual metabolic diversity of hyperalkaliphilic microbial communities associated with subterranean serpentinization at The Cedars. *ISME J* **11**, 2584-2598 (2017).

7 Nobu, M. K. *et al.* Unique H_2-utilizing lithotrophy in serpentinite-hosted systems. *ISME J* **17,** 95–104 (2023).

8 Holmes, D. E. *et al.* A membrane-bound cytochrome enables *Methanosarcina acetivorans* to conserve energy from extracellular electron transfer. *MBio* **10,** e00789-19 (2019).

9 Holmes, D. E., Zhou, J., Ueki, T., Woodard, T. & Lovley, D. R. Mechanisms for electron uptake by *Methanosarcina acetivorans* during direct interspecies electron transfer. *MBio* **12**, e02344-21 (2021).

10 McGlynn, S. E., Chadwick, G. L., Kempes, C. P. & Orphan, V. J. Single cell activity reveals direct electron transfer in methanotrophic consortia. *Nature* **526**, 531-535 (2015).

11 Chadwick, G. L. *et al.* Comparative genomics reveals electron transfer and syntrophic mechanisms differentiating methanotrophic and methanogenic archaea. *Plos Biol* **20**, e3001508 (2022).

12 Smith, J. A. *et al.* Mechanisms involved in Fe(III) respiration by the hyperthermophilic archaeon Ferroglobus placidus. *Appl Environ Microbiol* **81**, 2735-2744 (2015).

13 Laso-Perez, R. *et al.* Thermophilic archaea activate butane via alkyl-coenzyme M formation. *Nature* **539**, 396-401 (2016).

14 Baquero, D. P. *et al.* Extracellular cytochrome nanowires appear to be ubiquitous in prokaryotes. *Cell* **186,** 13, 2853 - 2864.e8 (2023).

15 Pohlschroder, M. & Esquivel, R. N. Archaeal type IV pili and their involvement in biofilm formation. *Front Microbiol* **6**, 190 (2015).

16 Lovley, D. R. & Walker, D. J. F. Geobacter Protein Nanowires. *Front Microbiol* **10**, 2078

(2019).

17 Vargas, M. *et al.* Aromatic amino acids required for pili conductivity and long-range extracellular electron transport in Geobacter sulfurreducens. *MBio* **4**, e00105-00113 (2013).

18 Kjeldsen, K. U. *et al.* On the evolution and physiology of cable bacteria. *Proc Natl Acad Sci USA* **116**, 19116-19125 (2019).

19 Nickel, L. *et al.* Two CRISPR-Cas systems in *Methanosarcina mazei* strain Go1 display common processing features despite belonging to different types I and III. *RNA Biol* **10**, 779-791 (2013).

20 Aravind, L. & Koonin, E. V. DNA polymerase beta-like nucleotidyltransferase superfamily: identification of three new families, classification and evolutionary history. *Nuc Acid Res* **27**, 1609-1618 (1999).

21 Klenk, H. P. *et al.* The complete genome sequence of the hyperthermophilic, sulphate-reducing archaeon *Archaeoglobus fulgidus*. *Nature* **390**, 364-370 (1997).

22 Feng, X., Wang, Y., Zubin, R. & Wang, F. Core metabolic features and hot origin of *Bathyarchaeota*. *Engineering* **5**, 498-504 (2019).

23 Vanwonterghem, I. *et al.* Methylotrophic methanogenesis discovered in the archaeal phylum *Verstraetearchaeota*. *Nat Microbiol* **1**, 16170 (2016).
