# Supplementary material for: A non-methanogenic archaeon within the order Methanocellales
Source: Nat Commun. 2024 Jun 13;15:4858. doi: 10.1038/s41467-024-48185-5 (PMC11176372; doi:10.1038/s41467-024-48185-5)
Supplement: Supplementary file 3 — Description of Additional Supplementary Files [file 41467_2024_48185_MOESM3_ESM.pdf]

Description of Additional Supplementary Files:

Supplementary Data 1: Stats of MAGs related to Met12 in this study.

Supplementary Data 2: Stats of archaeal genomes used in this study.

Supplementary Data 3: Summary of MS ortholog clusters.

Supplementary Data 4: Number of homolog families between microbes based on MS orthoMCL clusters.

Supplementary Data 5: Functional annotation and in situ gene expression levels of CDSs in Met12 MAG.

Supplementary Data 6: Close relatives of MmcX of Met12 that was highly expressed in the Cedars serpentinization setting.

Supplementary Data 7: Predicted 3D structure of MmcX of Met12.
