# Supplementary material for: A non-methanogenic archaeon within the order Methanocellales
Source: Nat Commun. 2024 Jun 13;15:4858. doi: 10.1038/s41467-024-48185-5 (PMC11176372; doi:10.1038/s41467-024-48185-5)
Supplement: Supplementary file 11 — Reporting Summary [file 41467_2024_48185_MOESM11_ESM.pdf]

nature portfolio

Corresponding author(s): Shino Suzuki and Shun'ichi Ishii

Last updated by author(s): 2023/03/11

Reporting Summary

Nature Portfolio wishes to improve the reproducibility of the work that we publish. This form provides structure for consistency and transparency in reporting. For further information on Nature Portfolio policies, see our Editorial Policies and the Editorial Policy Checklist.

Statistics

For all statistical analyses, confirm that the following items are present in the figure legend, table legend, main text, or Methods section.

n/a	Confirmed	
☐	☒	The exact sample size (*n*) for each experimental group/condition, given as a discrete number and unit of measurement
☒	☐	A statement on whether measurements were taken from distinct samples or whether the same sample was measured repeatedly
☒	☐	The statistical test(s) used AND whether they are one- or two-sided<br>*Only common tests should be described solely by name; describe more complex techniques in the Methods section.*
☒	☐	A description of all covariates tested
☒	☐	A description of any assumptions or corrections, such as tests of normality and adjustment for multiple comparisons
☐	☒	A full description of the statistical parameters including central tendency (e.g. means) or other basic estimates (e.g. regression coefficient) AND variation (e.g. standard deviation) or associated estimates of uncertainty (e.g. confidence intervals)
☒	☐	For null hypothesis testing, the test statistic (e.g. *F*, *t*, *r*) with confidence intervals, effect sizes, degrees of freedom and *P* value noted<br>*Give P values as exact values whenever suitable.*
☒	☐	For Bayesian analysis, information on the choice of priors and Markov chain Monte Carlo settings
☒	☐	For hierarchical and complex designs, identification of the appropriate level for tests and full reporting of outcomes
☒	☐	Estimates of effect sizes (e.g. Cohen's *d*, Pearson's *r*), indicating how they were calculated

Our web collection on statistics for biologists contains articles on many of the points above.

Software and code

Policy information about availability of computer code

Data collection	Genbank, SRA, DRA, ARB SILVA, GTDB, arCOG, KO, IMG, KEGG pathway database, PDB
Data analysis	CLC Genomic Workbench v8.6, CLC Genome Finishing Module, BLAST Ring image generator, KEGG Automatic Annotation Server, BLAST, GhostKOARA, TMHMM, KBase, OrthoMCL, COUNT, iTOL, PyMOL, AlphaFold2, ColabFold, MUSCLE, RaxML,

For manuscripts utilizing custom algorithms or software that are central to the research but not yet described in published literature, software must be made available to editors and reviewers. We strongly encourage code deposition in a community repository (e.g. GitHub). See the Nature Portfolio guidelines for submitting code & software for further information.

Data

Policy information about availability of data

All manuscripts must include a data availability statement. This statement should provide the following information, where applicable:

- Accession codes, unique identifiers, or web links for publicly available datasets
- A description of any restrictions on data availability
- For clinical datasets or third party data, please ensure that the statement adheres to our policy

All unassembled sequences related to this study have been deposited in the NCBI Sequence Read Archive under accession numbers SRX17443947, SRX17443946, SRX17443944, SRX17443945, and SRX17428102 on BioProject PRJNA351917 [https://www.ncbi.nlm.nih.gov/bioproject/PRJNA351917]. The circulated genome data of MAG Met12 has been submitted in the NCBI GenBank under accession number CP017966 [https://www.ncbi.nlm.nih.gov/nuccore/2647574341]. The detailed

data generated in this study are provided in the Supplementary Data or Source Data files. Raw analytical data of Met12 CDSs generated in this study are provided in the Supplementary Data 5, while the source data of the orthologous analyses for the comparative genomes are provided in the Supplementary Data 3.

Research involving human participants, their data, or biological material

Policy information about studies with human participants or human data. See also policy information about sex, gender (identity/presentation), and sexual orientation and race, ethnicity and racism.

Reporting on sex and gender	n/a
Reporting on race, ethnicity, or other socially relevant groupings	n/a
Population characteristics	n/a
Recruitment	n/a
Ethics oversight	n/a

Note that full information on the approval of the study protocol must also be provided in the manuscript.

Field-specific reporting

Please select the one below that is the best fit for your research. If you are not sure, read the appropriate sections before making your selection.

☐ Life sciences ☐ Behavioural & social sciences ☒ Ecological, evolutionary & environmental sciences

For a reference copy of the document with all sections, see nature.com/documents/nr-reporting-summary-flat.pdf

Ecological, evolutionary & environmental sciences study design

All studies must disclose on these points even when the disclosure is negative.

Study description	New findings related to the Met12, an archaeon in the order Methanocellales detected from serpentinized springs in The Cedars, California. We used environmental samples, but this study is not a quantitative study.
Research sample	We focus on single archaeal organism in the order Methanocellales with in microbial communities.
Sampling strategy	Water filtration. The sample size (about 1000L of water) was determined to collect sufficient numbers of cells for metagenomic and metatranscruptomic analysis. No statistical methods for sample size calculations were not performed.
Data collection	Shino Suzuki collected the cell samples from the field by using in-line filter system. DNA and RNA extraction was performed in the Lab.
Timing and spatial scale	Samples were collected on September 29th in 2011 at GPS1 and BS5, June 15th in 2012 at GPS1 and BS5, and Sep 29th 2014 in GPS1, BS5 and BS5sc
Data exclusions	All data was used for the analysis
Reproducibility	Reproducibility was confirmed in the samples collected at three different years, from three different water sources.
Randomization	Randomization is irrelevant since our target is a specific organism and the study is not quantitative.
Blinding	Not applicable

Did the study involve field work? ☒ Yes ☐ No

Field work, collection and transport

Field conditions	Water temperature is around 18 degree C, pH is around 12, and Eh is around -650 mV.n/a
Location	Barnes Spring 5 (BS5) (elevation 282 m, N: 38°37.282’, W: 123°7.987’) and Grotto Pool Spring 1 (GPS1) (elevation 273 m, N: 38° 37.268’ W: 123°8.014’),

Access & import/export	Land owners allowed us to collect samples.
Disturbance	No disturbance was caused by the study.

Reporting for specific materials, systems and methods

We require information from authors about some types of materials, experimental systems and methods used in many studies. Here, indicate whether each material, system or method listed is relevant to your study. If you are not sure if a list item applies to your research, read the appropriate section before selecting a response.

Materials & experimental systems

n/a	Involved in the study
☒	☐ Antibodies
☒	☐ Eukaryotic cell lines
☒	☐ Palaeontology and archaeology
☒	☐ Animals and other organisms
☒	☐ Clinical data
☒	☐ Dual use research of concern
☒	☐ Plants

Methods

n/a	Involved in the study
☒	☐ ChIP-seq
☒	☐ Flow cytometry
☒	☐ MRI-based neuroimaging

Plants

Seed stocks	n/a
Novel plant genotypes	n/a
Authentication	n/a
